# Prediabetes/diabetes screening strategy at the periodontal clinic

**DOI:** 10.1002/cre2.338

**Published:** 2020-12-10

**Authors:** Andreas Grigoriadis, Ismo T. Räisänen, Pirjo Pärnänen, Taina Tervahartiala, Timo Sorsa, Dimitra Sakellari

**Affiliations:** ^1^ Department of Preventive Dentistry, Periodontology and Implant Biology Dental School, Aristotle University of Thessaloniki Thessaloniki Greece; ^2^ 424 General Military Training Hospital Thessaloniki Greece; ^3^ Department of Oral and Maxillofacial Diseases, Head and Neck Center University of Helsinki and Helsinki University Hospital Helsinki Finland; ^4^ Division of Periodontology, Department of Dental Medicine Karolinska Institutet Stockholm Sweden

**Keywords:** diabetes, diagnosis, matrix metalloproteinase

## Abstract

**Objective:**

The aim of the study was to propose an efficient chairside clinical strategy for the identification of undiagnosed hyperglycaemia in periodontal clinics.

**Material and methods:**

Α chairside system was used for assessment of glycated hemoglobin 1c (HbA1c) and active Matrix Metalloproteinase‐8 levels (aMMP‐8) were analyzed by immunotest in patients (*n* = 150) who fulfilled the criteria for screening of the Centers for Disease Control and Prevention. Full‐mouth periodontal parameters were assessed and various data such as Body Mass Index (BMI), smoking and education were recorded.

**Results:**

Thirty‐one patients out of 150 tested were found with unknown hyperglycaemia (20.7%). Regarding sex, education, parent with diabetes, normal BMI, smoking, age ≥45 years and prior testing for diabetes, no differences were observed between subjects displaying HbA1c < 5.7 and ≥5.7% (Pearson's Chi‐square test, *p* > .05). Subgroups differed regarding BMI (kg/m^2^), tooth count, percentages of 4 and 5 mm pockets (Mann–Whitney and *z*‐test, *p* < .05). The diagnostic performance for HbA1c ≥5.7 was tested by Receiving Operator Characteristic curves and Areas Under the Curve (AUC) for the following: age ≥ 45 years and BMI (AUC 0.651, *p* = .010), the above and aMMP‐8 (AUC 0.660, *p* = .006), age ≥ 45 years, BMI and Stage of Periodontitis (AUC 0.711, *p* < .001) and age ≥ 45 years, BMI, aMMP‐8 and stage of periodontitis (AUC 0.713, *p* < .001).

**Conclusions:**

Findings of the study suggest that the combination of stage of periodontitis, increasing age, BMI and aMMP‐8, without chairside HbA1c assessment appears to be a viable screening strategy for referring dental patients for testing for prediabetes/diabetes.

## 1 INTRODUCTION

Type 2 diabetes mellitus (DMT2) has become a global pandemic, leading to significant morbidity, mortality and financial healthcare issues (A.D.A., 2020). It has been estimated that 425 millions of individuals (20–79 years old) worldwide had diabetes in 2017 and this number is expected to rise into 629 millions by the year 2045 (Ogurtsova et al., 2017). Type 2 diabetes is often unrecognized, as it is asymptomatic during the initial stage of the disease. The global percentage of undiagnosed diabetes is alarming, and estimated to be 212.4 millions of adults in 2017, mainly from low and middle income countries (Cho et al., 2018). Prediabetes, defined as hyperglycaemia which is below the pathologic threshold but very close to it [Glycated hemoglobin 1c (HbA1c) 5.7–6.4%, and/or fasting plasma glucose (FPG) 100 mg/dL to 125 mg/dL] almost always precedes type 2 diabetes (C.D.C., 2017). However, as it has been solidly shown by randomized controlled clinical trials, lifestyle interventions are effective in preventing the progression of prediabetes to diabetes (Baker, Simpson, Lloyd, Bauman, & Singh, 2011; Howells, Musaddaq, McKay, & Majeed, 2016).

Given the above‐mentioned facts, early detection and timely intervention especially regarding the stage of prediabetes are of cardinal importance. Several studies have already investigated the effectiveness of diabetes screening in dental settings, applying various methods of patient selection and identification of hyperglycaemia (Barasch et al., 2012; Bossart et al., 2016; Franck, Stolberg, Bilich, & Payne, 2014; Genco et al., 2014; Grigoriadis et al., 2019; Herman, Taylor, Jacobson, Burke, & Brown, 2015; Holm et al., 2016; Lalla, Cheng, Kunzel, Burkett, & Lamster, 2013; Lalla, Kunzel, Burkett, Cheng, & Lamster, 2011; Li, Williams, & Douglass, 2011; Rosedale & Strauss, 2012; Strauss et al., 2010). Although these studies are heterogeneous with regards to criteria for patient selection and the technique applied for prediabetes/diabetes assessment, the general conclusion is that early diagnosis of prediabetes/diabetes is feasible by dentists, especially in periodontally compromised patients.

In parallel, periodontal pathology is known to upregulate pro‐inflammatory mediators such as tissue destructive matrix metalloproteinases (MMPs) including neutrophil collagenase known as collagenase‐2 (MMP‐8) in inflamed gingiva and oral fluids and an effective chairside test for active MMP‐8 and not the latent form is available for clinical praxis (Grigoriadis et al., 2019; Rathnayake et al., 2013; Ryan, Ramamurthy, Sorsa, & Golub, 1999; Safkan‐Seppälä, Sorsa, Tervahartiala, Beklen, & Konttinen, 2006; Sorsa et al., 1992; Sorsa et al., 2020).

The aim of the present study is to propose a chairside point‐of‐care (PoC) clinical strategy applied in patients attending periodontal clinics for the identification of undiagnosed hyperglycaemia.

## 2 MATERIALS AND METHODS

### 2.1 Subject sample

The minimally required sample size for identifying subjects with undiagnosed diabetes (*n* = 139) was calculated according to the estimated percentage of undiagnosed diabetes in Europe (10%) and by applying the relevant statistical equation as described before (Mataftsi, Koukos, & Sakellari, 2019). Consecutive periodontal patients who visited the Department of Periodontology, Dental School, Aristotle University, Thessaloniki, Greece, and the Periodontal Department of 424 General Army Hospital, Thessaloniki, Greece, formed the sample pool of 731 possible participants. The self‐assessed questionnaire proposed by the CDC (Centres for Disease Control and Prevention, USA) (“Centres for Disease Control and Prevention. Prediabetes Screening Test. National Diabetes Prevention Programme. Available at https://www.cdc.gov/diabetes/prevention/pdf/prediabetestest.pdf,”) was used to identify 150 patients being at high risk for developing diabetes mellitus (score > 9) and these subjects were included in the study (Supplementary Table 1). Participants signed an informed consent and the study was conducted according to the protocol outlined by the Research Committee, Aristotle University of Thessaloniki, Greece, and approved by the Ethical Committee of the School of Dentistry (protocol number #64, 06/12/2018). All procedures performed in the present study involving human participants were in accordance with the ethical standards of the institutional and/or national research committee and with the 1964 Helsinki declaration and its later amendments or comparable ethical standards.

### 2.2 Clinical procedures

In subjects who fulfilled the CDC criteria for developing diabetes type 2, the Cobas® b101 (Roche Diagnostics, Hoffmann La Roche, Mannheim, Germany) in vitro diagnostic test system for determination of HbA1c levels was applied. The system determines the amount of HbA1c in human capillary blood by photometric transmission measurement. The method has been standardized against the IFCC (International Federation of Clinical Chemists) reference method (Zhang et al., 2018). This diagnostic test also provides, at the same time, values of free glucose, when HbA1c is above 4.9%.

After this assessment, patients were instructed to provide an oral rinse in order to quantitate Matrix metalloproteinase (MMP)‐8 (neutrophil collagenase‐2) levels in its active form (aMMP‐8) by the chair‐side/Point of Care (POC) PerioSafe® immunotest, combined by the digital reader ORALyzer® according to the manufacturer's instructions (Dentognostics GmbH—Jena, Germany). It has been shown that aMMP‐8 levels >20 ng/mL are indicative of actual periodontal tissue destruction (Räisänen et al., 2018; Räisänen et al., 2019; Sorsa et al., 2016; Sorsa, Gieselmann, Arweiler, & Hernández, 2017) and therefore were considered as a threshold for active proinflammatory and tissue destructive events .

Several parameters including Body Mass Index (BMI), age, level of education, and smoking were also recorded. Periodontal examination included clinical measurement of Probing Depth (PD), Clinical Attachment Level (CAL), Bleeding on Probing (BOP) and Plaque Levels in six surfaces of each tooth, excluding third molars by using an automated probe (Florida Probe, Florida Probe Corporation, Gainesville, FL, USA) by one calibrated examiner (A.G.). All patients were classified by the 2018 classification of periodontal diseases (Papapanou et al., 2018).

All subjects identified with hyperglycaemia (HbA1c ≥ 5.7%) were strongly advised to contact their physician for further consultation, and laboratory tests. Participants were also asked about the ease and convenience of the procedure and whether they would repeat it at the dental clinic.

### 2.3 Statistical methods

Statistical analyses were performed with the SPSS Base 25.0. Statistical Software Package (SPSS Inc., IBM, Chicago, IL, USA). Data analysis was performed, and figures plotted with statistical software. Patient characteristics and their association with prediabetes and periodontal condition were tested by Pearson's Chi‐square test, Kruskal–Wallis *H* test, Welch's *t*‐test, and Mann–Whitney *U* test (Table 1). After a significant Kruskal–Wallis *H* test, Dunn–Bonferroni test was used for pairwise post hoc comparisons. Normality assumption for parametric tests was assessed graphically with a histogram and a Q–Q plot, and numerically with the Shapiro–Wilk test. All continuous variables (in prediabetes and periodontal condition calculations) in Table 1 were non‐normal.

**TABLE 1:** Patients characteristics according to HbA1c levels and periodontal condition

Patients data		HbA1c levels			Periodontal condition				
		HbA1c < 5.7	HbA1c ≥ 5.7	*p‐*Value	Healthy (N)	Stage I (N)	Stage II (N)	Stage III (N)	*p‐*Value
Sex	Women	59	17	.602	11	14	39	12	**.003**
	Men	60	14		20	1	42	11	
Education	Elementary	2	1	.077	0	1	2	0	**<.001**
	Middle	49	21		2	8	41	19	
	Technical school	4	0		0	0	3	1	
	University	51	8		20	5	31	3	
	Post graduate	13	1		9	1	4	0	
Parent with diabetes	Yes	44	10	.626	9	6	33	6	.470
	No	75	21		22	9	48	17	
Body mass index (kg/m^2^)	Mean ± SD	29.62 ± 4.58	31.9 ± 5.5	.033					
Smoking	No	77	23	.394	25	7	55	13	**.015**
	Yes	37	8		3	8	24	10	
	Electronic	5	0		3	0	2	0	
Age ≥ 45 years	Yes	91	27	.198	14	14	70	20	**<.001**
	No	28	4		17	1	11	3	
Prediabetes (HbA1c ≥ 5.7)	Yes	—	—	—	2	2	17	10	**.009**
	No				29	13	64	13	
Prior testing for diabetes	Yes	24	3	.176	2	5	18	2	.057
	No	95	28		29	10	63	21	
Tooth count	Mean ± SD (%)	24.86 ± 2.96	23.39 ± 3.54	**.026**	26.81 ± 1.89	24.20 ± 2.52	24.41 ± 2.86	22.22 ± 3.87	**<.001**
4 mm pocket count	Mean ± SD (%)	30.76 ± 33.56	49.06 ± 39.65	**.019**	4.55 ± 5.89	10.40 ± 10.15	40.20 ± 33.60	70.78 ± 34.40	**<.001**
5 mm pocket count	Mean ± SD (%)	14.87 ± 24.26	25.16 ± 8.90	*.014	0.77 ± 1.73	1.93 ± 2.71	18.09 ± 23.75	44.83 ± 31.84	**<.001**
6 mm pocket count	Mean ± SD (%)	5.09 ± 10.85	8.16 ± 13.40	**.064**	0.26 ± 0.77	0.40 ± 0.91	4.80 ± 8.20	19.83 ± 19.19	**<.001**

*Note:* *p*‐values for variables sex, education, parent with diabetes, normal Body Mass Index, smoking, age ≥ 45 years, prediabetes (HbA1c ≥ 5.7), and prior testing for diabetes was calculated by Pearson's Chi‐square test (asymptotic, 2‐sided) for prediabetes and periodontal condition. *p‐*Values for BMI, tooth count, 4 mm pocket count, 5 mm pocket count and 6 mm pocket count were calculated by Mann–Whitney *U* test (exact, 2‐sided) for prediabetes and Kruskal–Wallis *H* test (asymptotic, 2‐sided) for periodontal condition; Welch's *t*‐test agreed with Mann–Whitney *U* test and Kruskal–Wallis *H* test for all continuous variables in accepting and declining *H*_0_, except for variable 5 mm pocket count (*p* value 0.075, marked with *) in prediabetes subgroup calculation.

For clinical parameters, PD, CAL, BOP and Plaque Levels, indicators of Descriptive Statistics were used, such as mean and SD for each subgroup, with the patient as the observational unit. Differences in clinical parameters were sought in subgroups formed by aMMP‐8 <20 ng/mL and >20 ng/mL, and HbA1c <5.7 and ≥5.7 by applying the Mann–Whitney *U* test (Table 2).

**TABLE 2:** Periodontal parameters in investigated subgroups

	PD (mm)	CAL (mm)	ΒOP	Plaque
	Mean ± SD	Mean ± SD	Mean ± SD	Mean ± SD
HbA1c <5.7 [*n* = 119]	**2.81 ± 0.78**	**3.18 ± 1.06**	**0.50 ± 0.24**	0.47 ± 0.28
HbA1c ≥5.7 [*n* = 31]	**3.20 ± 0.94**	**3.54 ± 1.20**	**0.62 ± 0.25**	0.52 ± 0.27
aMMP‐8 < 20 [*n* = 98]	**2.68 ± 0.67**	**3.04 ± 0.97**	**0.49 ± 0.25**	**0.43 ± 0.25**
aMMP‐8 > 20 [*n* = 52]	**3.30 ± 0.94**	**3,77 ± 1.17**	**0.60 ± 0.22**	**0.57 ± 0.29**
HBA1c < 5.7&aMMP8 < 20 [*n* = 81]	**2.63 ± 0.65**	**3.00 ± 0.99**	**0.47 ± 0.25**	**0.44 ± 0.26**
HBA1c > 5.7&aMMP8 > 20 [*n* = 14]	**3.57 ± 1.03**	**4.36 ± 1.19**	**0.69 ± 0.20**	**0.63 ± 0.25**

*Note:* Differences between subgroups are indicated by bold lettering, Mann–Whitney test *p* < .05.

Abbreviations: aMMP‐8: active matrix metalloproteinase −8; BOP: Bleeding on probing; CAL: clinical attachment level; HBA1c: glycated hemoglobin A1c; PD: probing depth.

Firth's bias‐reduced logistic regression was used for exploring the association between the active MMP‐8 (aMMP‐8) point‐of‐care test (ORALyzer®) and prediabetes, because there was a low prevalence for many variables. This leads to so called complete separation, which is a statistical challenge occurring when the dependent variable separates one (or more than one) variable completely. As a result, the estimates for the independent variables cannot be obtained, as the maximum likelihood does not exist. A common approach to this problem is to use Firth's method with a penalized maximum likelihood estimation (Heinze & Schemper, 2002). Receiver Operating Characteristic (ROC) curves were created using logistic regression models calculated by Firth's method and their predicted probabilities. The ROC curves were analyzed by Youden's index (Youden, 1950) to find efficient cut‐off points for the models. Based on this cut‐off and prediabetes prevalence, models were compared according to their performance in prediabetes diagnostics (Supplementary Table 2).

A two‐sided *p* value <.05 was considered statistically significant in this study.

## 3 RESULTS

Thirty‐one patients out of 150 tested were found to have HbA1c ≥ 5.7% (20.7%), 24 of which had HbA1c 5.7–6.4%, indicating undiagnosed prediabetes (16%), and seven displayed HbA1c ≥ 6.5% (range 6.8–8.9%) indicating undiagnosed diabetes (4.7%). The remaining participants (119 patients) had HbA1c < 5.7%.

Patients characteristics according to HbA1c levels and periodontal condition are presented in Table 1. Regarding variables sex, education, parent with diabetes, smoking, age ≥45 years and prior testing for diabetes, no statistically significant differences were observed between subjects displaying HbA1c levels <5.7 and ≥5.7 (Pearson's Chi‐square test, *p* > .05). In contrast, these subgroups differed regarding BMI (kg/m^2^), tooth count, percentages of 4 mm and 5 mm pockets, with subjects in the hyperglycaemia group exhibiting lower number of teeth, and higher numbers of 4 and 5 mm pockets (Mann–Whitney *U* test, *p* < .05). Also, a larger proportion of subjects with HbA1c levels ≥5.7 exhibited Stage III periodontitis compared to periodontally healthy individuals (*p* < .05 *z*‐test with Bonferroni correction).

Parameters which statistically significantly affected the periodontal condition of participants were sex, education, smoking, age ≥45 years and HbA1c levels ≥5.7 (Pearson's Chi‐square test, *p* < .05), while the tooth count decreased and number of pockets 4, 5 and 6 mm gradually increased according to the Stage of periodontitis (Kruskal–Wallis *H* test, *p* < .001). Further, periodontally healthy patients had significantly higher number of teeth compared to Stage I, II and III patients (Dunn–Bonferroni test, *p* < .05, *p* < .001, *p* < .001, respectively). Similarly, the number of 4 mm, 5 mm and 6 mm pocket counts were significantly different in all five pairwise comparisons between healthy and Stage II and III (Dunn–Bonferroni test, *p* < .05), but not between healthy and Stage I patients (Dunn–Bonferroni test, *p* > .05).

Periodontal parameters and differences between subgroups are depicted in Table 2.

Participants were stratified according to HbA1c levels, aMMP‐8 levels above and below the threshold of 20 ng/mL and the combined presence or not of both HbA1c levels ≥5.7 and aMMP‐8 >20 ng/mL. Subjects with HbA1c levels ≥5.7, aMMP‐8 levels >20 ng/mL and the combined presence of elevated levels exhibited statistically significantly higher values of BOP, PD and CAL, compared to participants with HbA1c levels <5.7, aMMP‐8 levels <20 ng/mL and the combined absence of elevated levels for these parameters respectively (Mann–Whitney *U* test, *p* < .05). Plaque levels were significantly elevated in the same groups except for HbA1c (Mann–Whitney U test, *p* < .05).

**FIGURE 1:**
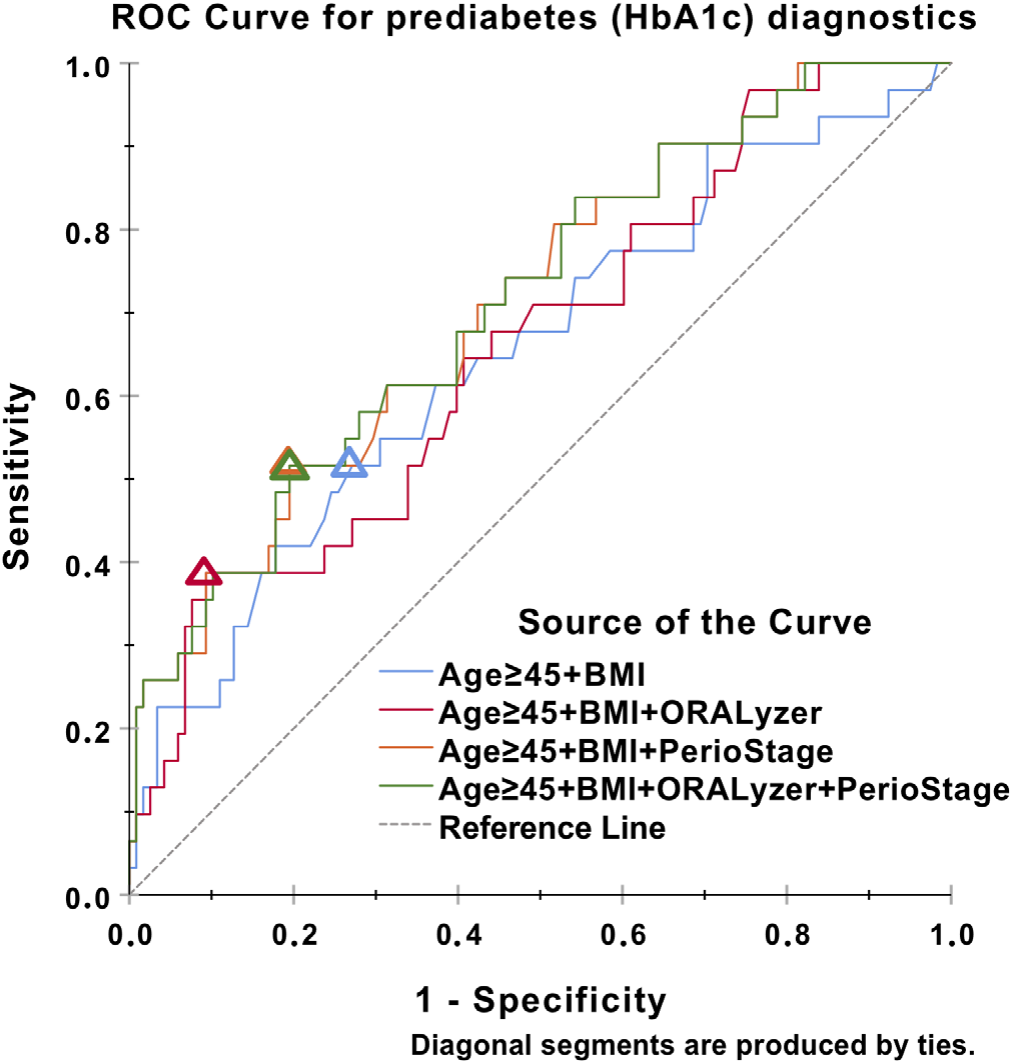
ROC curve for prediabetes (HbA1c) diagnostics based on four Firth's bias‐reduced logistic regression models built by aMMP‐8 levels measured by ORALyzer, Periodontitis stage (PerioStage), and prediabetes risk factors BMI and Age ≥ 45 years. Optimal cut‐offs (Youden's index) on the ROC curves for each model denoted by the triangles. ROC curve for prediabetes (HbA1c) diagnostics based on four Firth's bias‐reduced logistic regression models built by aMMP‐8 levels measured by ORALyzer, Periodontitis stage (PerioStage), and prediabetes risk factors BMI and Age ≥ 45 years. Optimal cut‐offs (Youden's index) on the ROC curves for each model denoted by the triangles

The diagnostic performance for HbA1c ≥ 5.7 was tested for a number of parameters and their combinations with the calculated optimal cut‐offs (Figure 1 and Supplementary Table 2). In univariable Firth's biased reduced logistic regression, aMMP‐8 levels as measured by ORALyzer, Periodontitis stage and BMI were significantly associated with HbA1c ≥ 5.7 (*p* = .0497, *p* = .0012 and *p* = .0216, respectively), while age ≥ 45 years was not.

ROC curves were constructed and Areas Under the Curve (AUC) calculated for the following combinations: Model 1: age ≥ 45 years and BMI, Model 2: age ≥ 45 years, BMI and aMMP‐8 > 20 ng/mL, Model 3: age ≥ 45 years, BMI and Stage of Periodontitis and finally Model 4: age ≥ 45 years, BMI, aMMP‐8 > 20 ng/mL and Stage of Periodontitis. For base model, Model 1, AUC was 0.651 (*p* = .010), while for Model 2 AUC was 0.660 (*p* = .006). Model 3 depicted AUC 0.711 (*p* < .001) and Model 4 AUC was .713 (*p* < 0.001).

All participants reported that the procedure was easy, convenient and patient‐friendly and that they would repeat it, if required.

## 4 DISCUSSION

The present study demonstrates that performing a chairside HbA1c measurement in the dental settings can depict subjects with undiagnosed hyperglycaemia, since the screening strategy managed to identify 24 patients (16% of the sample) with undiagnosed prediabetes and seven patients (4.7% of the sample) with undiagnosed diabetes. Altogether, subjects with previously undiagnosed hyperglycaemia were 20.7% of the 150 participants of the study. This fact allows for early diagnosis of prediabetes/diabetes by referring these subjects to medical doctors for further investigation. It is reminded that the above subject sample consisted of individuals fulfilling the criteria for further screening of the CDC (“Centres for Disease Control and Prevention [CDC]. Prediabetes Screening Test. National Diabetes Prevention Programme. Available at https://www.cdc.gov/diabetes/prevention/pdf/prediabetestest.pdf,”). In the absence of the feasibility of performing chairside HbA1c assessment in the dental clinic, a Point‐of‐Care aMMP‐8 mouth‐rinse test can improve the screening ability of validated questionnaires especially in periodontally diseased subjects.

According to the CDC, an estimated 33.9% of U.S. adults aged 18 years or older (84.1 million people) had prediabetes in 2015, based on their fasting glucose or HbA1c levels, with adults aged 65 years depicting prediabetes at an especially high percentage (48.3%) (C.D.C., 2017). Among adults with prediabetes, 11.6% reported being told by a health professional that they had this condition, while age‐adjusted data for 2011–2014 indicated that more men (36.6%) than women (29.3%) had prediabetes and that prevalence of prediabetes was similar among racial and ethnic groups. In the US alone, it was estimated that USD 44 billion was spent on healthcare due to prediabetes (Dall et al., 2014).

The prevalence of DMT2 in Greece remains high, and according to recent data (Liatis et al., 2016) it accounts for 7.0% of the population (with 8.2% prevalence of DMT2 for people ≥15 years of age). On the other hand, prediabetes prevalence is not well studied, with some estimates from regional studies raising it to around 22% of the adult population (Makrilakis et al., 2011). Recently published data regarding 139 Greek subjects with periodontal disease who also fulfilled the CDC criteria for screening and were chairside‐assessed for HbA1c with the Cobas® b101 system have shown that almost 25% of the subjects tested had unknown hyperglycaemia (Mataftsi et al., 2019). In addition, subjects with HbA1c ≥ 5.7% displayed higher proportions of sites with clinical attachment loss >5 mm. These findings suggested that periodontal patients, especially those with bigger than normal BMI and waist circumference, are a target group worth screening for diabetes at the dental clinic (Mataftsi et al., 2019).

In the present study, participants were recruited irrelevant of periodontal condition and the percentage of not previously known hyperglycaemia was 20.7%. Both percentages are in agreement with the estimates for prediabetes in Greece as reported in studies from the medical field.

The recent consensus report of the joint workshop of the International Diabetes Federation and the European Federation of Periodontology reports that dentists dealing with patients without a diagnosis of diabetes are encouraged to apply screening methods and assess their risk for having diabetes, in order to refer to a physician for further testing identified subjects (Sanz et al., 2018). In fact, the importance of validated questionnaires has been shown in a number of studies and they can be used with reasonable accuracy for prediabetes/diabetes screening (Bang et al., 2009; Herman, Smith, Thompson, Engelgau, & Aubert, 1995; Poltavskiy, Kim, & Bang, 2016; Rolka et al., 2001). This approach is certainly low‐cost and therefore suitable for large‐scale assessments both in clinical and community settings especially in low income countries. However, albeit this well established approach as shown in the current study according to model 1 (Figure 1), it is suggested that in the absence of chairside assessment of glycated hemoglobin A1c, an aMMP‐8 chairside test could act—apart from periodontal inflammation—as a surrogate marker in order to refer patients for further evaluation by their physicians. This fact can contribute to the overall worldwide effort to limit the “pandemic” of diabetes type 2 along with several studies investigating the possible participation of dental practitioners to alert patients, by referring to medical practitioners and resulting to an early diagnosis and/or treatment.

In fact, within the last years there are a number of studies investigating the feasibility of diabetes screening in dental settings. In these studies hyperglycaemia was identified either by chairside automated analyzers or in collaboration with a laboratory (Baker et al., 2011; Barasch et al., 2012; Bossart et al., 2016; Franck et al., 2014; Genco et al., 2014; Grigoriadis et al., 2019; Herman et al., 2015; Holm et al., 2016; Lalla et al., 2011; Lalla et al., 2013; Li et al., 2011; Rosedale & Strauss, 2012; Strauss et al., 2010).

Although these studies vary significantly regarding their design, sample size, age, racial and ethnic background of participants tested, they all conclude that it is feasible to screen dental patients for diabetes at the occasion of the dental visit (opportunistic screening). Noteworthy, that it has been reported that patients tend to visit their dentist on a more regular basis compared to their physician (Glick & Greenberg, 2005). In the present study, HbA1c and aMMP‐8 chairside measurements were applied in patients attending periodontal clinics who were in high risk of developing diabetes, according to the CDC questionnaire, irrelevant of their periodontal condition (health or inflammation). Since it is not clear which diagnostic laboratory or chairside method is the most appropriate for identifying people with prediabetes, in the current study we have chosen to apply HbA1c assessment, a reliable test considered to reflect the state of blood glucose levels over a time period (Sequeira & Poppitt, 2017) and more convenient compared to FPG or oral glucose tolerance test since no fasting is required (A.D.A., 2020). Known disadvantages of this specific test include availability of test mainly in developed countries (A.D.A., 2020) and the possibility of effects of ethnicity or hemoglobin variants (Barry et al., 2017).

According to the findings and in agreement with the literature subjects with HbA1c > 5.7 exhibited statistically significant differences in terms of clinical parameters of periodontal disease (Tables 1 and 2) thus underlying the contribution of hyperglycaemia to inflammation of periodontal tissues. This fact was also shown, when Periodontitis stage according to the 2018 classification (Papapanou et al., 2018) was integrated in Receiver Operator Curves for diagnosing hyperglycaemia (HbA1c ≥ 5.7%) either in combination with age ≥ 45 years and BMI, or in combination with the above and aMMP‐8 values (ORALyzer®). In both cases as shown in Supplementary Table 2 and Figure 1 the diagnostic performance for screening for prediabetes/diabetes is quite satisfactory, especially when compared to using only the known prediabetes/diabetes risk factors age ≥ 45 years and BMI. In other words, both cases help to reduce the amount of false positive findings and unnecessary referrals of patients to further prediabetes/diabetes testings. Thus, increasing stage of periodontitis, increasing age and splachnic obesity as well as elevated aMMP‐8 levels in mouth‐rinse appear to be important factors for a periodontist to encourage the patient for screening for diabetes. (Supplementary Table 2, Figure 1). In the case of the POC aMMP‐8 test, albeit its low cost and convenience, it is surely not intended for massively screening populations, but application at the dental office, especially for periodontitis patients as shown in Figure 1, can strengthen the reasons for a dentist to strongly recommend to a patient to get further checked by a physician and receive recommended instructions/treatment. It should be mentioned that this quantitative Point‐of‐Care test is commercially available and in use for online and real‐time diagnosis and treatment monitoring of periodontitis (Alassiri et al., 2018; Grigoriadis et al., 2019; Johnson et al., 2016; Lorenz et al., 2017; Nwhator et al., 2014; Räisänen et al., 2018; Räisänen et al., 2019; Raivisto, Sorsa, Räisänen, et al., 2020; Sorsa et al., 2017), while assessment for HbA1c as applied in the present and other studies, is not yet easily feasible for a dental practice. A recent systematic review has shown that the dental workforce can be beneficially engaged in screening for prediabetes/diabetes but further clinical trials are required in order to optimize risk assessment protocols and strategies (Yonel et al., 2020).

Besides, the incorporation of validated biomarkers will improve diagnostic accuracy and assessment of stage and grade of the new Periodontitis classification system by Tonetti et al (Tonetti, Greenwell, & Kornman, 2018). and recent data have shown that the aMMP‐8 mouth‐rinse test can offer this possibility (Sorsa et al., 2020). This fact, is corroborated by findings of the present study, since, as shown in Table 2, the subgroup of participants with aMMP‐8 above 20 ng/mL displayed statistically significant differences in clinical parameters of periodontal disease compared to subjects with ORALyzer® values below this threshold.

Taken collectively, findings of the present study suggest that the combination of periodontitis, increasing age, BMI and aMMP‐8, when the use of chairside methods of HbA1c assessment is not available appears to be a viable screening strategy for correctly referring dental patients for further testing for prediabetes/diabetes by their physicians.

## 5 CONCLUSIONS

In line with the previously published relevant reports, this study provides further supporting and extending evidence that the periodontal clinic is ideal for opportunistic screening for prediabetes/diabetes. Utilization of point‐of care technology, that is, mouth‐rinse aMMP‐8 PoC assay enhances the ability of practitioners to contribute to the global effort for early diagnosis of prediabetes and/or T2 diabetes mellitus.

## CONFLICT OF INTEREST

Timo Sorsa is the inventor of US‐patent 10 488 415 B2 and a Japanese patent 2016‐554676. Other authors report no conflicts of interest related to this study. The funders had no role in the design of the study; in the collection, analyses or interpretation of data; in the writing of the manuscript, or in the decision to publish the results.

## Supporting information


**Supplementary Table 1** Self‐assessed questionnaire proposed by the Centers for Disease Control and Prevention for the identification of pre‐diabetes (https://www.cdc.Prediabetestest.pdf)

**Supplementary Table 2** The diagnostic performance of Firth's bias‐reduced logistic regression models for predicting HbA1c ≥ 5.7%. Their efficient cut‐offs were calculated by Youden's index, and 2 × 2 confusion matrix were constructed by this cut‐off point.

## Data Availability

The data that support the findings of this study are available from the corresponding author upon reasonable request
