# Supplementary material for: Prediabetes/diabetes screening strategy at the periodontal clinic
Source: Clin Exp Dent Res. 2020 Dec 10;7(1):85–92. doi: 10.1002/cre2.338 (PMC7853879; doi:10.1002/cre2.338)
Supplement: Supplementary file 1 — Supplementary Table 1 Self‐assessed questionnaire proposed by the Centers for Disease Control and Prevention for the identification of pre‐diabetes (https://www.cdc.Prediabetestest.pdf) [file CRE2-7-85-s001.docx]

**Supplementary Table 1.** Self-assessed questionnaire proposed by the Centers for Disease Control and Prevention for the identification of pre-diabetes (https://www.cdc.Prediabetestest.pdf)

| Are you a woman who has had a baby weighing more than 4.08 kg at birth? | Yes (1) | No (0) |
| --- | --- | --- |
| Do you have a sister or brother with diabetes? | Yes (1) | No (0) |
| Do you have a parent with diabetes? | Yes (1) | No (0) |
| \| Do you weigh more than the weight listed for your height, according to the BMI chart? \| \| --- \| \| (i.e. is your BMI bigger than normal?) \| | Yes (5) | No (0) |
| Are you younger than 65 years of age and get little or no exercise on a typical day? | Yes (5) | No (0) |
| Are you between 45 and 64 years of age? | Yes (5) | No (0) |
| Are you 65 years of age or older? | Yes (9) | No (0) |
