# Supplementary material for: Prediabetes/diabetes screening strategy at the periodontal clinic
Source: Clin Exp Dent Res. 2020 Dec 10;7(1):85–92. doi: 10.1002/cre2.338 (PMC7853879; doi:10.1002/cre2.338)
Supplement: Supplementary file 2 — Supplementary Table 2 The diagnostic performance of Firth's bias‐reduced logistic regression models for predicting HbA1c ≥ 5.7%. Their efficient cut‐offs were calculated by Youden's index, and 2 × 2 confusion matrix were constructed by this cut‐off point. [file CRE2-7-85-s002.docx]

**Supplementary Table 2.** The diagnostic performance of Firth’s bias-reduced logistic regression models for predicting HbA1c≥5.7%. Their efficient cut-offs were calculated by Youden’s index, and2x2 confusion matrix were constructed by this cut-off point.

| HbA1c ~ ORALyzer, cut-off 26.35 ng/mL | HbA1c≥5.7 | HbA1c<5.7 | OR | Se(%) | Sp(%) | FN(%) | FP(%) | Acc(%) | MCC |
| --- | --- | --- | --- | --- | --- | --- | --- | --- | --- |
| positive | 14 | 26 | 2,91 | 45,2 | 78,0 | 15,6 | 65,0 | 71,1 | 0,21 |
| negative | 17 | 92 |  |  |  |  |  |  |  |
|  |  |  |  |  |  |  |  |  |  |
| HbA1c ~ PerioStage, cut-off Stage III | HbA1c≥5.7 | HbA1c<5.7 | OR | Se(%) | Sp(%) | FN(%) | FP(%) | Acc(%) | MCC |
| Stage III | 10 | 13 | 3,85 | 32,3 | 89,0 | 16,7 | 56,5 | 77,2 | 0,24 |
| N-Stage II | 21 | 105 |  |  |  |  |  |  |  |
|  |  |  |  |  |  |  |  |  |  |
| Model 1:  HbA1c ~ Age≥45+BMI, cut-off 0,25 | HbA1c≥5.7 | HbA1c<5.7 | OR | Se(%) | Sp(%) | FN(%) | FP(%) | Acc(%) | MCC |
| positive | 16 | 32 | 2,87 | 51,6 | 72,9 | 14,9 | 66,7 | 68,5 | 0,21 |
| negative | 15 | 86 |  |  |  |  |  |  |  |
|  |  |  |  |  |  |  |  |  |  |
| Model 2:  HbA1c ~ Age≥45+BMI+ORALyzer, cut-off 0.32 | HbA1c≥5.7 | HbA1c<5.7 | OR | Se(%) | Sp(%) | FN(%) | FP(%) | Acc(%) | MCC |
| positive | 12 | 11 | 6,14 | 38,7 | 90,7 | 15,1 | 47,8 | 79,9 | 0,33 |
| negative | 19 | 107 |  |  |  |  |  |  |  |
|  |  |  |  |  |  |  |  |  |  |
| Model 3:  Age≥45+BMI+PeriosStage, cut-off 0.27 | HbA1c≥5.7 | HbA1c<5.7 | OR | Se(%) | Sp(%) | FN(%) | FP(%) | Acc(%) | MCC |
| positive | 16 | 23 | 4,41 | 51,6 | 80,5 | 13,6 | 59,0 | 74,5 | 0,30 |
| negative | 15 | 95 |  |  |  |  |  |  |  |
|  |  |  |  |  |  |  |  |  |  |
| Model 4: Age≥45+BMI+PeriosStage+ORALyzer, cut-off 0.27 | HbA1c≥5.7 | HbA1c<5.7 | OR | Se(%) | Sp(%) | FN(%) | FP(%) | Acc(%) | MCC |
| positive | 16 | 23 | 4,41 | 51,6 | 80,5 | 13,6 | 59,0 | 74,5 | 0,30 |
| negative | 15 | 95 |  |  |  |  |  |  |  |

Age≥45: age ≥45 years; BMI: body mass index; PerioStage: periodontitis stage; OR: odds ratio; Se: sensitivity; Sp: specificity; FN: false negatives; FP: false positives; Acc: accuracy; Matthew’s correlation coefficient
